# Selective capturing of phenolic derivative by a binary metal oxide microcubes for its detection

**DOI:** 10.1038/s41598-019-55891-4

**Published:** 2019-12-17

**Authors:** Mohammed Muzibur Rahman

**Affiliations:** Department of Chemistry, King Abdulaziz University, Jeddah, 21589 P.O. Box 80203, Saudi Arabia

**Keywords:** Environmental monitoring, Two-dimensional materials

## Abstract

Development of highly efficient and potential material for toxic p-nitrophenol is an important design for sensitive detection of hazardous species from ecology and environment. Here it is developed, an efficient as well as selective of p-nitrophenol using binary material by electrochemical performances, including good linearity, lower detection limit, good stability, higher reproducibility and extreme sensitivity. The prepared electrode was fabricated by immobilization of SnO_2_/CdO microcubes (MCs) with conducting coating binders by using well-known glassy carbon electrode (GCE). The proposed MCs with SnO_2_/CdO were well-functionalized and prepared by facile hydrothermal technique. The general instrumentation namely, FTIR, UV/vis, FESEM, XPS, TEM, EDS, and powder XRD were employed for the morphological evaluation of the prepared doped MCs, structural, optical and elemental analyses. The large dynamic range (LDR) from 1.0 to 0.01 mM with 0.13 pM detection limit (S/N = 3), limit of quantification (LOQ; 0.43 pM), and an excellent sensitivity of 7.12 µAµM^−1^cm^−2^ were exhibited by the fabricated binary material based on SnO_2_/CdO MCs for selective p-nitrophenol capturing. In shortly, the SnO_2_/CdO MCs can be employed as an efficient electron mediator with binary materials fabricated GCE for capturing the p-nitrophenol at ultra-trace amounts. Then the binary synthesized material of SnO_2_/CdO MCs is used as potential and sensitive sensor layer by stable electrochemical approach for sensitive capturing of toxic p-nitrophenol from environmental samples.

## Introduction

The nitrophenol are usually toxic and bio-refractory organic substances, which are diversified modern large-scale application of the solvent to produce varieties of significantly utilized devices, including pharmaceutical and chemicals, pesticide, diverse dyes, fungicide, insecticide and aniline^1^. Then the nitrophenol has been listed as hazardous by EPA (Environmental Protection Agency) owing to the high toxicity toward the ecology, environment, and severely affected by the human health, animal, plants and living organisms^2–4^. Particularly for the human, nitrophenol is responsible for cancer producing, generative poisonousness and tumors of the urinary tract^5,6^. Due to the highly solubility nature of nitrophenol, this substance is usually existed in industrial wastewater, surface water and marine aquatic environment^7^. Consequently, this is prime demand for the development of reliable and portable device to the monitoring in industrial working places, environment and as well the public health sectors for detection and quantify of toxic phenolic derivatives to avoid the water contamination and unwanted harmful effect on human health and environment^8^. The distinctive conventional technique exclusively are applied for the detection of nitrophenol such as spectrophotometry, fluorescence, GC-MS, capillary electrophoresis, and HPLC^9–13^, but these tradition methods are not user friendly. To conventional detection of nitrophenol, the electrochemical method has user friendly features such as, economic viability, easy to operate, rapidity, and quicker response time, highly selective, and finally extremely sensitive in room conditions^14^.

Due to the wider band-gap, the semiconductor metal oxides such as TiO_2_, ZnO, SnO_2_, Fe_2_O_3_, etc were exclusively employed as highly-potential electron mediator for the fabrication of efficient chemical sensors^15^. According to Wu *et al*., the p-nitrophenol material was fabricated by α-MnO_2_ nanotubes, which displayed an excellent affinity up to 0.019 µAµM^−1^ cm^−2^ with remarkable low limits of detection as 0.1 mM^16^. Another literature claimed that the materials based on Mn_2_O_3_/ZnO are less affinity, and it is about 4.6667 μAcm^−2^μM^−1^ and the lower limit of detection are 0.83 ± 0.2 nM^17^. The carbon nanotube is an important sensing element for detection of hazardous and toxic spices in the field of electrochemistry. It is becoming more popular up to date from the first invention^18–21^. Based on experimental work of Wu *et al*., the 4-nitrophenol material synthesized with carbon nanotube in the presence of phthalocyanine cobalt(II), showed a remarkable low limit of detection with lower detecting range^22^. Another efficient nitrophenol adsorbent material was produced by PDDA, which is fabricated by grapheme (PDDA-G), are displayed outstanding data both in the detection limit and linear range of concentration^23^.

Because of the extreme toxicity of p-nitrophenol to the human health, a reliable and efficient technique is applied with potential materials. In this study, we have fabricated binary SnO_2_/CdO consisting with MCs and GCE electrode accumulation mode for an efficient monitoring of p-nitrophenol with the electrochemical approach. The expeditions of p-nitrophenol by SnO_2_/CdO MCs, which was kept on the top of GCE which was connected by adhesive. The broad range studies were done toward the fabrication of p-nitrophenol detecting electrode to keep safe of the ecology and environment. All other experimental parameters were measured systematically and discussed in detail in the proceeding sections. The electrochemical sensing was developed and the data with binary conjugate material was exhibited and found the higher sensitivity in the optimum conditions. The extracted data were outstanding on sensor development, and the binary material was excellent to detect the toxic p-nitrophenol substance significantly with novel binary conjugated material as SnO_2_/CdO MCs sensor probe in connection with Nafion/GCE.

## Materials and Methods

### Materials

For this work, the required chemicals were purchased from the Sigma-Aldrich, such as ammonium hydroxide, 4-aminophenol, 4-methoxyphenol, 5% nafion (in ethanolic solution), p-nitrophenol, monosodium phosphate acetone, 2-nitrophenol, 3-methoxyphenol, bisphenol, ethanol, hydrazine, methanol, and disodium phosphate. The NICOLET iS50 FTIR (Madison, WI, USA) as well as 300 UV-vis (Thermo Scientific) was used to analysis the functional properties and band-gap energies of synthesized calcined SnO_2_/CdO MCs material. For investigating of the practical binding energies (eV) of elemental composition such as oxygen (O), cadmium (Cd), and tin (Sn), XPS system was used as well for SnO_2_/CdO MCs (Thermo scientific, K-α1 1066). The properties of prepared microcubes such as molecular arrangements, elemental analysis of morphological evaluation, and shape as well as sizes of SnO_2_/CdO MCs were investigated in details by FESEM (JSM-7600F, Japan). For crystallinity microcubes material, the prepared SnO_2_/CdO MCs were also totally investigated with powder XRD instrument. The current versus potential relation was done for the detection of p-nitrophenol in the selective potential range. The synthesized GCE with SnO_2_/CdO MCs was investigated as working electrode.

### Synthesis of low-dimensional SnO_2_/CdO MCs by hydrothermal approach

For the doped microcubes preparation, reactants tin chloride (SnCl_4_), cadmium chloride (CdCl_2_) and reducing agent, ammonium hydroxides (NH_4_OH) were initially used as reactants into the reactor by facile hydrothermal technique. Here, hydrothermal method is generally applied for the preparation of transition metal undoped/doped semiconductor nanostructure materials. The finally prepared doped nanomaterials were obtained smaller in size like fine-powder for the phase formation after precipitation. Accordingly, as reactor cell was poured by the DI water and reactant precursor CdCl_2_ and SnCl_4_ were systematically dissolved into this reactor flask for starting the reaction. Then the reactor was kept on the stage, which stirring continuously by magnetic stirring system. Reducing agent, NH_4_OH was systematically added into the reactor of reactant solution to maintain the pH in the basic region. The solution was heated at 150 °C under continuous oven for well mixing and completes the reaction. Then the reaction is put into the air to complete dry and settle the precipitate. The solid precipitate was collected and washed with water, acetone and ethanol to remove any inorganic and organic contaminants systematically. Finally, the dried precipitate of SnO_2_/CdO crystal was calcined at 510.0 °C for 6 hours. The calcine material was fully grounded and made the fine-powder and start for the further evaluation to characterize the prepared sample properly. Here, the calcined material was fully characterized in terms of optical, functional, structural, morphological, elemental analyses by using UV/vis, FTIR, XRD, EDS, XPS, and FESEM, etc. Finally, the fabricated nanomaterial was coated onto the GCE and applied for the p-nitrophenol sensor development by using electrochemical approaches at room conditions.

1 $${{\rm{NH}}}_{4}{{\rm{OH}}}_{({\rm{s}})}\to {{\rm{NH}}}_{4({\rm{aq}})}^{+}+{{\rm{OH}}}_{({\rm{aq}})}^{-}$$

2 $${{\rm{CdCl}}}_{2({\rm{s}})}\to {{\rm{Cd}}}_{({\rm{aq}})}^{2+}+2{{\rm{Cl}}}_{({\rm{aq}})}^{-}$$

3 $${{\rm{SnCl}}}_{4({\rm{s}})}\to {{\rm{Sn}}}_{({\rm{aq}})}^{4+}+4{{\rm{Cl}}}_{({\rm{aq}})}^{-}$$

4 $$\begin{array}{c}{{\rm{NH}}}_{4({\rm{aq}})}^{+}+6{{\rm{OH}}}_{({\rm{aq}})}^{-}+{{\rm{Sn}}}_{({\rm{aq}})}^{4+}+{{\rm{Cd}}}_{({\rm{aq}})}^{2+}+{{\rm{Cl}}}_{({\rm{aq}})}^{-}\to {{\rm{Sn}}({\rm{OH}})}_{4({\rm{aq}})}\downarrow +{{\rm{Cd}}({\rm{OH}})}_{2({\rm{aq}})}\downarrow \\ \,+\,{{\rm{NH}}}_{4}{{\rm{Cl}}}_{({\rm{aq}})}\end{array}$$

5 $${{\rm{Sn}}({\rm{OH}})}_{4({\rm{aq}})}\downarrow +{{\rm{Cd}}({\rm{OH}})}_{2({\rm{aq}})}\downarrow \to {{\rm{SnO}}}_{2}-{{\rm{CdO}}}_{({\rm{s}})}\downarrow +3{{\rm{H}}}_{2}{{\rm{O}}}_{({\rm{aq}})}$$

**Figure 1:**
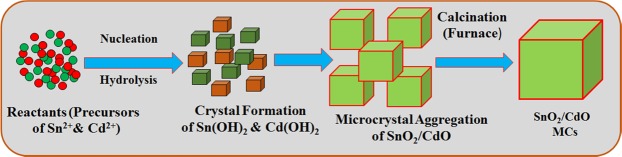
Step-wise fabrication of the binary SnO_2_/CdO MCs using well-known hydrothermal approach.

Accordingly, the Eqs. (1–5), the proposed reactions are accomplished and made completion after vigorous shaking. During the crystal formation of SnO_2_/CdO in the reactor, the pH was functioned the substantial role in the reaction mixtures. After pH adjusted over 10, the reactant CdCl_2_ in solution is begun to hydrolysis and converted to hydroxide of cadmium ions (Eq. (4)) accordingly. So, the NH_4_OH is the responsible for supplying the hydroxide (OH^−^) as well as generated in the basic area. At that time, Cd^2+^ and OH^−^ ions were enhanced significantly, where Cd(OH)_2_ is started to form doped nuclei. Based on the reaction of (iii) and (iv), increasing of Sn^4+^ ions in the basic area, the nanocrystal with Cd(OH)_2_ termed to be higher due to small activation energy of Cd(OH)_2_. Owing to greater Sn^4+^ ions remaining in the solution phase, Cd(OH)_2_, Sn(OH)_4,_ hydroxide crystals are begun to accumulated microcubic structure in solid phase. Microcubic crystal of heated SnO_2_/CdO was similar in growth, which was described in former reports^24,25^. After that, the whole solution was washed by water and organic solvent (ethanol and acetone) consecutively. Later, the prepared nanocrystal product was dried fully at ambient conditions for 12 h and then calcined. Hence, by Ostwald-ripening technique for crystal microcubes gain, initially SnO_2_/CdO MCs nucleus was formed by self- and mutual-heterogeneous gathering and after that, the MCs was re-gathered to form crystalline SnO_2_/CdO MCs. The internal molecular as well as atomic arrangement of microcrystal crystal was formed via Vander-Waals forces. The formation mechanism of SnO_2_/CdO MCs is shown in Fig. 1. Then the produced SnO_2_/CdO MCs were totally determined (optical, elemental, morphological, functional, and structural, etc.) by various conventional methods and finally used for the capturing of p-nitrophenol at room conditions after coating with 5% nafion onto GCE electrode.

### Preparation of GCE with calcined SnO_2_/CdO MCs

For the preparation of buffer solution at pH 7.0, the equi-molar of disodium phosphate (0.2 M Na_2_HPO_4_) and monosodium (0.2 M NaH_2_PO_4_) were added in water. The working electrode of SnO_2_/CdO MCs was prepared by gently coated with the GCE. A few drop of 5% nafion solution was dropped as required. The fabricated GCE was kept at 35 °C for 1 h to form in uniform. The different concentration of p-nitrophenol solution (full concentration range: 1.0 pM ~ 1.0 mM) was made, which was employed in the accumulated cell to p-nitrophenol. In p-nitrophenol capturing methodology, the sensitivity of the binary SnO_2_/CdO MCs was investigated. The limit of detection (DL) as well as the linear range (LDR) was defined accordingly. Electrochemical current response is measured with SnO_2_/CdO microcubes fabricated GCE sensor probe at a room condition by the electrochemical method.

## Results and Discussion

### Optical and structural analyses of binary SnO_2_/CdO MCs

**Figure 2:**
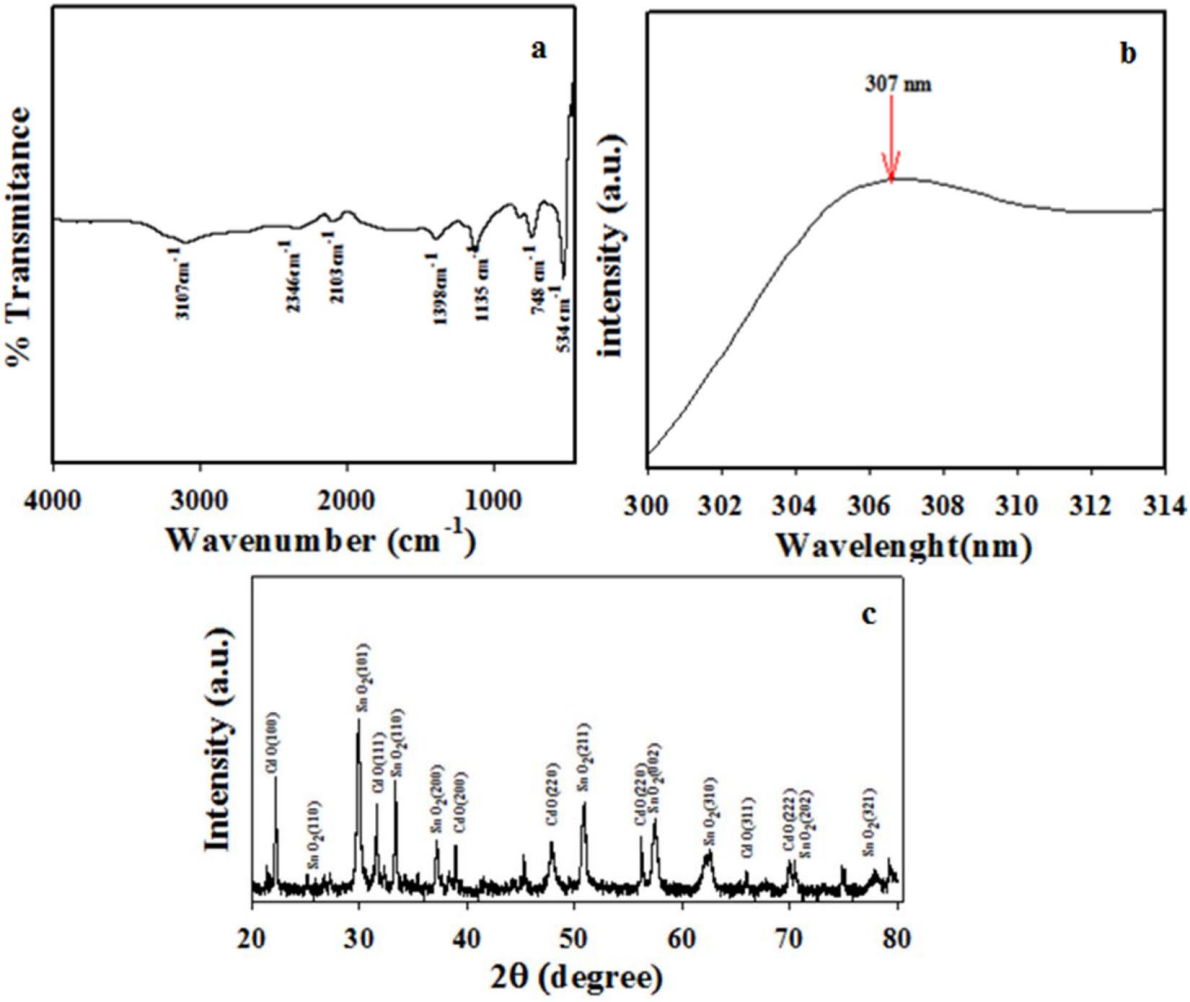
Optical and structural analyses using well-known tools of (**a**) FTIR, (**b**) UV-Vis. and (**c**) XRD to the SnO_2_/CdO microcubes.

The photo-sensitive is the vital measurable representative in the inspection of photo-catalytic property of the fabricated binary SnO_2_/CdO MCs. Then the proposed microcube materials were performed by UV-vis spectrophotometer because of the scanning affinity with the visible-light source. Also the energy is absorbed by the exterior electron from low to the high-energy area^26^. During the visible-light absorption by compound, a corresponding spectrum of UV-vis is attained which indicated the desired wavelength absorbed by atom of synthesized microcube material. The UV-vis investigation was operated from the area as selected from 400 to 800 nm at standard mode, and the data are shown in Fig. 2(b). The specific illustrated spectrum is displayed with an excessive and broad spectrum at 307 nm. This is similar band and accredited electronic crossing from low to the high energy level valence band of the binary SnO_2_/CdO MCs. Then the data is conceded to the visual band-gap of E_bg,_ is 4.04 eV. This can be defined as E_bg_ = 1240/λ_max_, where the E_bg_ is band gap and λ_max_ represents the maximum absorptive wave^24^.

The functional group in the prepared materials was analyzed by FTIR. The SnO_2_/CdO MCs is implemented for FTIR investigation, which is presented in Fig. 2(a). According to the Fig. 2(a), peaks at 534,748, 1135, 1398, 2103, 2346 and 3107 cm^−1^ were observed in the spectrum. As the metal oxide (M-O) band is observed in the lower region less than 1000 cm^−1^. Here, it is found 534 cm^−1^ and 748 cm^−1^ peaks, which are observed the stretching vibration of Sn-O-Sn oxygen^27^. Here the stretching modes of COOH group are found to be at 1135 and 1398 cm^−1^ ^28^. Here, hydroxyl (-OH) group is presented about the asymmetric and symmetric stretching vibrations at 3107, 2346 and 2103 cm^−1^ due to the adsorption of H_2_O molecules onto the surface of binary mixed nanomaterials^29,30^.

To clarify the doped nanomaterials crystallinity of binary mixed SnO_2_/CdO MCs, it was performed by XRD. In Fig. 2(c), the sharp peaks were exhibited in the XRD diffraction, which conveyed the information’s about that the synthesized SnO_2_/CdO MCs, which was associated of SnO_2_ and CdO. The diffracted peaks at (100), (111), (200), (220), (222) and (311) are directly matched from CdO indices (JCPDS No. 05-0640)^31–33^. Crystalline and face-centred cubic structure are found from this observation. The identical structural properties and phase information of CdO nanostructure are observed by Ravichandran *et al*.^34^, which is prepared by using co-precipitation technique. The nano-crystalline size of prepared CdO nanostructure is exhibited with the higher intense peaks for the crystal planes.

Similarly, many sharp peaks were displayed at (002), (101), (110), (112), (200), (202), (211), and (321), which were belonged to the crystalline phase of SnO_2_^35^ with the JCPDS No. 06-0395. The crystalline peaks were found to match with orthorhombic structure of SnO_2_ in the nanocomposites of CdO–SnO_2_ which are closely matched with JCPDS data (06-0395).

There is an arrangement to know crystallinity based on the XRD, and this was a co-relation with Scherer’s equation:

6 $${\rm{D}}=0.9{\rm{\lambda }}/({\rm{\beta }}\,\cos \,{\rm{\theta }})$$

where, the values of λ, β, θ are the wavelength (1.5418 Å), full-wave width at half-maximum, and diffracting-edge respectively, based on the highest peak^36^. From the the Scherer’s equation, it is calculated the particle sized as 1.7 µm. Consequently, the results indicated that the binary doped nanomaterial consist of CdO and SnO_2_ phases, confirming the formation of CdO–SnO_2_ nanostructures.

### Morphological and elemental analyses of binary SnO_2_/CdO MCs

**Figure 3:**
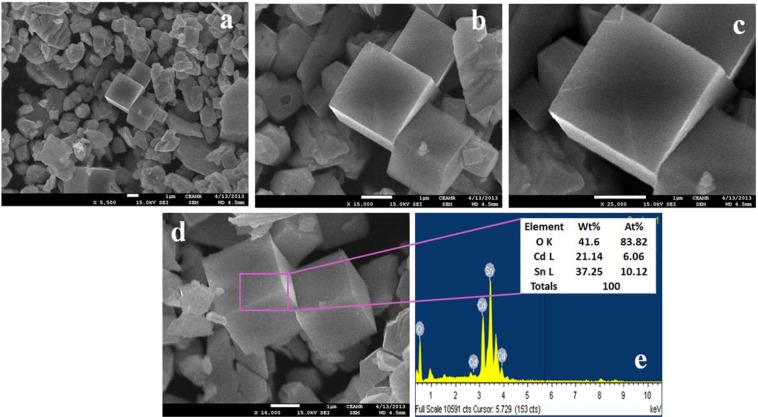
Morphological and elemental analysis. (**a**,**b**) The micrographs of FESEM and (**c**,**d**) mapping of different elements consisting of the binary SnO_2_/CdO/GCE MCs.

The FESEM images were captured to understand the structural moiety of the prepared SnO_2_/CdO MCs and the pictures are shown in Fig. 3(a,b). This is noted that an average cross-section of the microcubes is about 2.1 µm in the 1.5 to 2.3 µm. Then the materials are consisted with cubes in size and shape^37–39^, which was defined by EDS mapping as stated in Fig. 3(c,d). The consisting elements doped microcubes materials were found from these images. Based on the EDS mapping, tin (Sn), oxygen (O), and cadmium (Cd) were consisted of 41.6 wt% (O), 21.14 wt% (Cd), and 37.25 wt% (Sn). The mapping also clarified that no other elements are consisted in this MCs^24,40^.

**Figure 4:**
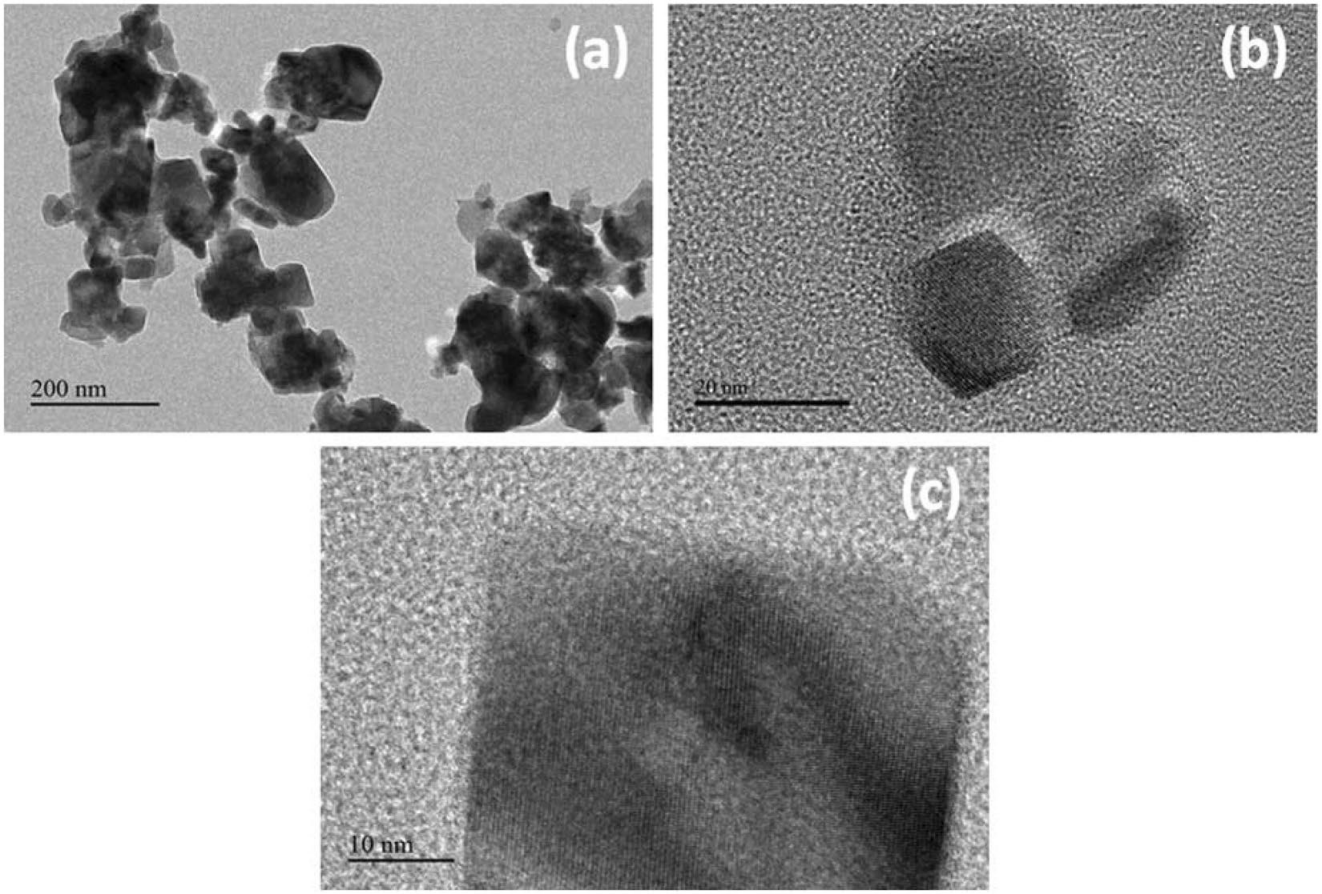
TEM analysis. (**a**–**c)** Low to high magnified TEM images of SnO_2_/CdO MCs.

Additionally, the NCs were also visualized using TEM (JEOL, Japan; Transmission Electron Microscope; TEM) operating at an accelerating voltage of 120 kV. Drops of the MCs suspensions were deposited on carbon-coated copper grids. Size-distributions were extracted from the TEM images and the mean diameters of cubes were measured. Here, it is measured the morphology for the binary SnO_2_/CdO MCs. A clear cube like shapes are observed on the images, which is represented in Fig. 4(a–c).

### Determination of binding energy of binary SnO_2_/CdO MCs

**Figure 5:**
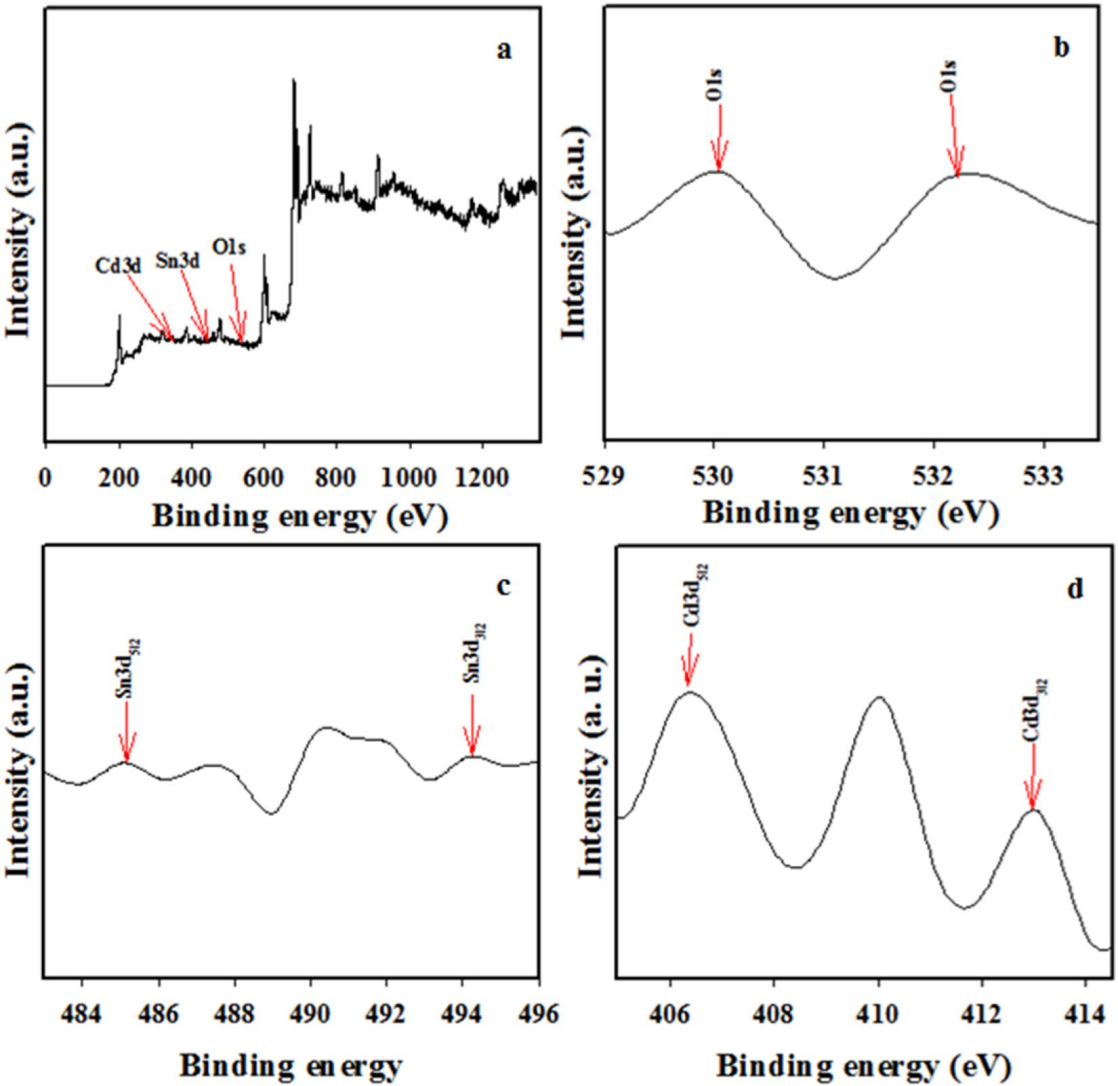
Evaluation of binding energy using XPS of the binary SnO_2_/CdO microcubes at ambient conditions, where. (**a**) Full spectrum, (**b**) O1s area, (**c**) spin-orbit Sn3d and (**d**) spin-orbit Cd_3d_ area.

The XPS was implemented to know the consisted matrices of the fabricated binary SnO_2_/CdO MCs, where the electrons are existed due to the kinetic energy in the sample based on the X-ray irradiation of the prepared materials. The prepared materials composition and reciprocal chemical structure are identified by the XPS^41^. The accumulated data are stated in Fig. 5. The data implied that three elements O, Sn and Cd are existed. The major two exact peaks in XPS spectra for Sn3d orbit in the sample of SnO_2_/CdO MCs and these are similar in nature, which clarified the similar oxidation state of Sn in the prepared materials. The binding energy for Sn3d_5/2_ and Sn3d_3/2_ corresponds to 485.08 and 494.08 eV and these are complied with oxidation Sn^4+^ in SnO_2_^42,43^. The data are illustrated in Fig. 5(c), where the O1s absorbance of SnO_2_/CdO MCs. The peak was certainly displayed clearly recognized, which was middle at 530.08 eV and was revealed of lattice O of SnO_2_^44,45^ depicted in Fig. 5(b). Figure 5(d) clarified the existence of Cd with similar two sharp peaks of Cd3d_5/2_ and Cd3d_3/2_ are also corresponded to 406.08 eV and 413.08 eV^46,47^.

### Detection of p-nitrophenol by binary SnO_2_/CdO MCs

**Figure 6:**
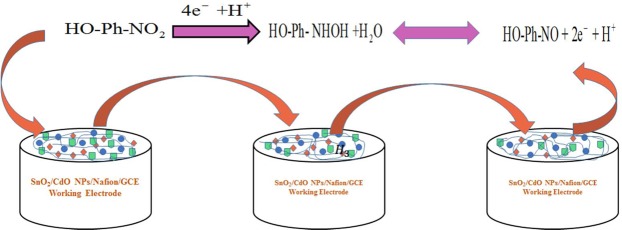
The possible mechanism for detection of p-nitrophenol by binary SnO_2_/CdO MCs/Nafion/GCE sensor probe.

The p-nitrophenol was detected by the constructed binary materials of SnO_2_/CdO MCs/Nafion/GCE under the defined buffer solution. The detection of p-nitrophenol using the SnO_2_/CdO MCs is the first attempt, and no report was indicated until today. The employed current vs potential volt was estimated at the p-nitrophenol sensing using with phosphate buffer mode by the SnO_2_/CdO MCs/Nafion/GCE electrode. This was enhanced remarkably in terms of accumulation of p-nitrophenol through the working electrode. The possible sensing mechanism of p-nitrophenol is shown in Fig. 6. In p-nitrophenol oxidation with fabricated electrode with SnO_2_/CdO MCs/Nafion/GCE assembly, the electron enhancement was displayed because of the applied current vs potential volt at suitable conditions. According to the proposed p-nitrophenol oxidation mechanism, at the beginning, p-nitrophenol was reduced to 4-hydroxylaminophenol and in the second step, the 4-hydroxylaminophenol was oxidized to 4-nitrosophenol followed by a consequent reversible reduction^48,49^, which were presented in Eqs. (7 and 8). The potential use of the binary SnO_2_/CdO MCs to p-nitrophenol detecting was demonstrated in Fig. 6. The following representation reveals and presented in below:

Glassy carbon electrode was coated using slurry of SnO_2_/CdO MCs and the constructed electrode was deployed to detection of p-nitrophenol analytes due to the oxidation in the buffer system, which is proposed and presented in below as per chemical reaction (Eqs. (7 and 8)). Based on the oxidation reaction of the targeted p-nitrophenol, electrons was discharged to the conduction band, which increased the electrochemical current with SnO_2_/CdO MCs binary material sensor probe.

7 $${\rm{HO}} \mbox{-} {\rm{Ph}} \mbox{-} {{\rm{NO}}}_{2}+4{{\rm{e}}}^{-}+{{\rm{H}}}^{+}\to {\rm{HO}} \mbox{-} {\rm{Ph}} \mbox{-} {\rm{NHOH}}+{{\rm{H}}}_{2}{\rm{O}}$$

8 $${\rm{HO}} \mbox{-} {\rm{Ph}} \mbox{-} {\rm{NHOH}}\leftrightarrow {\rm{HO}} \mbox{-} {\rm{Ph}} \mbox{-} {\rm{NO}}+2{{\rm{e}}}^{-}+{{\rm{H}}}^{+}$$

**Figure 7:**
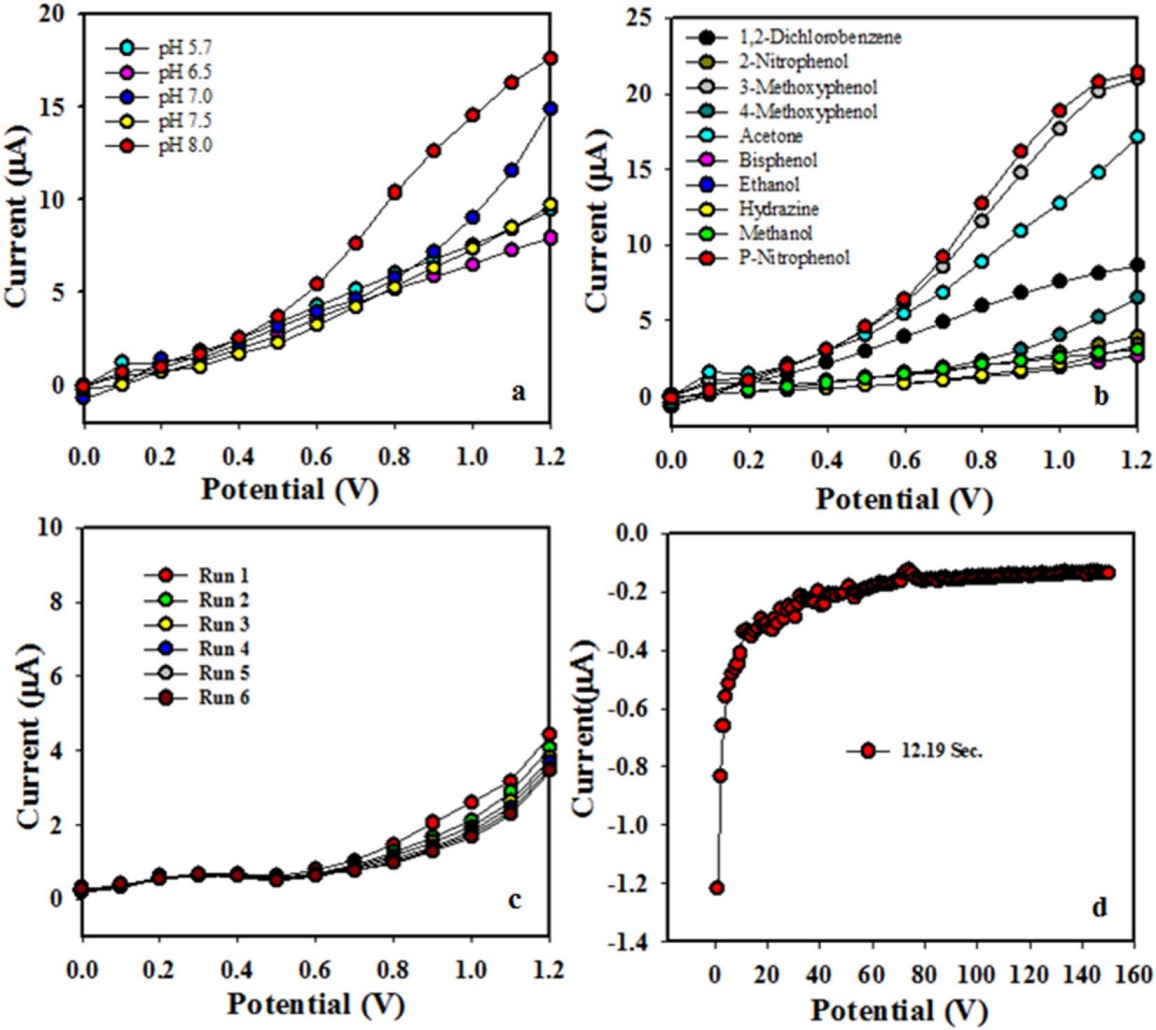
Optimization of different experimental parameters evaluation of the binary SnO_2_/CdO MCs based GCE electrode, (**a**) pH effect, (**b**) selectivity, (**c**) reproducibility, and (**d**) response time.

To define the solution pH, the binary electrode was deployed for the detection in broad pH ranges and the data clarified that the produced modified electrode with SnO_2_/CdO MCs was more responsive to pH 8.0 compared to other buffer systems as shown in Fig. 7(a) using I-V assessment at voltage range from 0 to +1.2 V. The selectivity was carried out using several similar classified chemicals to understand specificity of the binary material. According to the data presentation, the binary materials showed extreme selectivity towards the p-nitrophenol. Therefore, it was remarked that p-nitrophenol showed the maximum current towards the binary material assembly (SnO_2_/CdO MCs/GCE) compared to the others chemical reagents, which are presented in Fig. 7(b). In order to evaluate the reuses of SnO_2_/CdO MCs, the detection current was also assessed. The reproducibility data is shown in Fig. 7(c). The extreme reuses characteristic were observed even after several reuses cycles of the proposed binary materials. The measured RSD was 2.48%. The response time to the ultra-trace amount (1.0 µM) of p-nitrophenol by the binary SnO_2_/CdO MCs was an excellent result with 12.2 second in response time, which was achieved and presented in Fig. 7(d).

**Figure 8:**
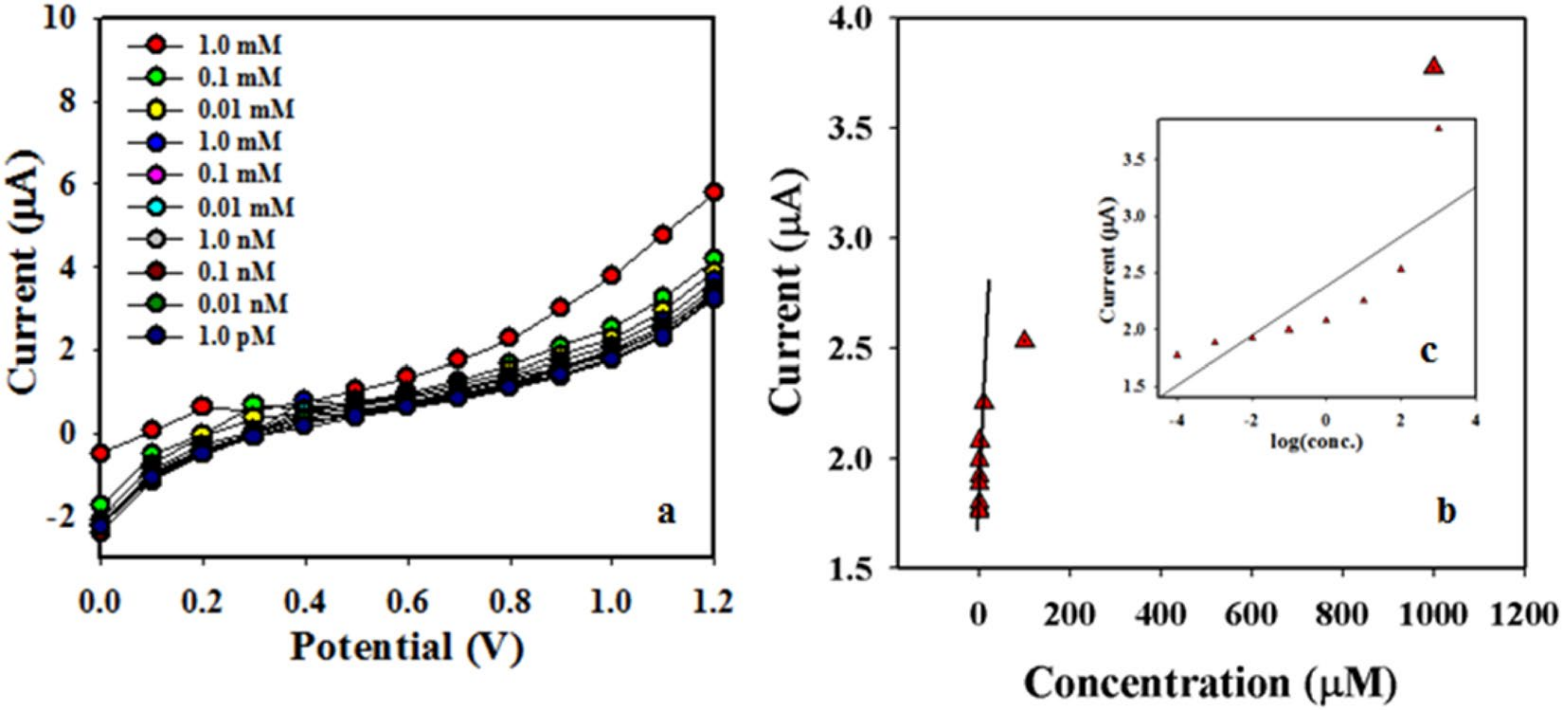
Determination of detection limit based on the (**a**) low to high amount of p-nitrophenol detecting by the binary SnO_2_/CdO MCs fabricated GCE and (**b**) linear dynamic range in the calibration graph according to log [p-nitrophenol amount] vs. current).

The working electrode consisted with binary SnO_2_/CdO MCs was responding to the various amounts of p-nitrophenol at ambient media. Then the I-V feedback of binary SnO_2_/CdO MCs was carried out based on the current vs potential with varying of p-nitrophenol. The data are depicted here in Fig. 8(a). Similarly, the detection limit was defined according to the linear range as plotted in Fig. 8(b). The data are clarified the high coefficient (linearity, r^2^: 0.99667), sensitivity (3N/S: 7.0886 µAµM^−1^cm^−2^), a linear dynamic range (from 1.0 pM to 0.01 mM), limit detection (LD: 0.13 pM), and limit of quantification (LOQ; 0.43 pM).

The current intensity was increased in the enhancement of the p-nitrophenol amount in the aqueous media. The identical trend is also reported for different toxin monitoring^16,50–52^. In a suitable protocol, the p-nitrophenol was involved to fewer amounts of surface sites of the binary SnO_2_/CdO MCs and oxidation process of the p-nitrophenol starts gradually. This is the main reason that p-nitrophenol can be detected from low to high range concentrations. The significant result was accomplished on the proposed working electrode of SnO_2_/CdO MCs/GCE assembly. Therefore, it can be summarized that the proposed binary material based on SnO_2_/CdO MCs sensor probe can detect the maximum towards the target p-nitrophenol. The resultant current value is observed maximum for p-nitrophenol compared to other compounds in identical condition by electrochemical method. This is also noted that the binary SnO_2_/CdO MCs has a quick response time with extreme sensitivity, then the present materials can exhibit extreme removal and excellent biocompatibility^22,50,52^.

### Potentiality in real samples

To use in practical application of the proposed binary SnO_2_/CdO MCs materials based sensor probe, the p-nitrophenol containing in real environmental and extracted waste samples was investigated. In this investigation, several waste samples were measured and presented in Table 1. The data clearly revealed that the binary SnO_2_/CdO MCs material based sensor probe was the suitable candidate for potential use in large-scale operation for the environmental and extracted samples for the safety of environmental and healthcare fields.

**Table 1:** Potential application of the binary SnO_2_/CdO MCs for p-nitrophenol capturing in environmental samples (recovery method).

Real Samples	Observed current (µA)				Average	Calculated Conc. (µM)	Recovery (%)	SD
	R1	R2	R3	R4				
Industrial effluents	0.93	0.81	0.79	0.74	0.81	0.39	97.8%	0.08
PC-baby-bottles	1.03	0.95	0.88	0.82	0.92	0.44	101.3%	0.08
PC-water-bottles	1.48	1.59	1.54	1.47	1.52	0.73	99.5%	0.06
PVC-food-containers	1.32	1.24	1.28	1.21	1.26	0.61	98.9%	0.05

*R = reading, SD = standard deviation.

## Conclusions

In conclusion, the doped SnO_2_/CdO microcube was fabricated using the hydrothermal approach at a low temperature, and the configurations of synthesized microcubes were characterized using SEM, EDS, FTIR, XPS and XRD. The binary material SnO_2_/CdO MCs was coated on GCE, which was displayed as an extreme and efficient electron mediator during oxidation of p-nitrophenol in phosphate buffer media. In the sensitive detection of p-nitrophenol even at ultra-trace amount, the proposed electrode was shown the highest current responses and good catalytic activity toward p-nitrophenol substance. Our synthesized p-nitrophenol sensitive binary material sensor probe has a lower limit of detection (DL) of 0.13 pM, with moderately higher sensitivity of 7.12025 µAµM^−1^ cm^−2^ and the LDR from 1.0 to 0.01 mM in the p-nitrophenol sensing at room conditions. Therefore, the proposed binary materials is introduced a new route for sustainable development of an efficient p-nitrophenol sensing probe by using doped nanostructure nanomaterials for the safety of environmental and healthcare fields.
